# Imaging-Based Diagnostic Approaches in Moyamoya Disease: A Scoping Review

**DOI:** 10.3390/jcm15062410

**Published:** 2026-03-21

**Authors:** Carlos Novillo-Solis, Micaela Salvador-Orbea, Andrea Morales-Acosta, Jose E. Leon-Rojas

**Affiliations:** 1 Escuela de Medicina, Universidad de Las Américas, Quito EC170124, Ecuador; carlos.novillo@udla.edu.ec (C.N.-S.); micaela.salvador@udla.edu.ec (M.S.-O.); 2 NeurALL Research Group, Quito EC170157, Ecuador; 3 Grupo de Investigación Bienestar, Salud y Sociedad, Escuela de Psicología y Educación, Universidad de Las Américas, Quito EC170124, Ecuador

**Keywords:** Moyamoya disease, neuroimaging, diagnostic imaging, cerebrovascular reserve, multimodal imaging

## Abstract

Moyamoya disease (MMD) is a chronic, progressive cerebrovascular disorder characterized by steno-occlusive changes in the intracranial internal carotid arteries and the development of fragile collateral networks. Imaging plays a pivotal role in diagnosis, disease staging, and management, yet the expanding range of available imaging modalities has resulted in heterogeneous evidence that remains difficult to synthesize. This scoping review aimed to systematically map and critically appraise imaging-based diagnostic approaches used in MMD, summarizing their diagnostic performance, clinical utility, and limitations. A comprehensive literature search was conducted across major databases, and original studies evaluating imaging modalities in human MMD were included. Thirty-three studies published between 1995 and 2023 were analyzed, encompassing digital subtraction angiography, magnetic resonance imaging and angiography, perfusion and functional MRI, computed tomography-based techniques, nuclear medicine, ultrasound, neurophysiological methods, and emerging artificial intelligence applications. Digital subtraction angiography remains the diagnostic reference standard, particularly for disease confirmation and surgical planning. However, noninvasive modalities provide critical complementary information. Magnetic resonance-based techniques offer multiparametric assessment of vascular morphology, hemodynamics, vessel wall pathology, and parenchymal injury. Computed tomography angiography and perfusion imaging provide accessible alternatives with high sensitivity for vascular changes, while functional and neurophysiological methods contribute additional hemodynamic and regional assessments. Artificial intelligence applications show promising diagnostic performance but remain in early validation stages. The evidence base is limited by methodological heterogeneity, inconsistent reference standards, incomplete reporting of diagnostic accuracy metrics, and a scarcity of longitudinal and multimodal studies. Collectively, the findings support a multimodal imaging strategy in MMD, integrating structural and functional information to inform diagnosis and management. Future research should prioritize standardized protocols, longitudinal designs, and clinically validated imaging biomarkers to enable evidence-based diagnostic pathways.

## 1. Introduction

Moyamoya disease (MMD) is a rare, chronic, and progressive cerebrovascular disorder characterized by bilateral or unilateral stenosis or occlusion of the terminal internal carotid arteries and their proximal branches, most commonly the anterior and middle cerebral arteries, with the development of a compensatory network of abnormal collateral vessels at the base of the brain [1,2,3,4,5,6]. The angiographic appearance of these fragile collaterals resembles a “puff of smoke,” which inspired the Japanese term “moyamoya” used to describe the disease [1,2,3,7]. Clinically, MMD is associated with a broad spectrum of neurological manifestations driven by cerebral hypoperfusion and collateral vessel fragility; these include ischemic stroke, intracerebral and intraventricular hemorrhage, transient ischemic attacks, seizures, epilepsy, cognitive impairment, and progressive neurological disability, affecting both pediatric and adult populations [1,4,7,8]. Disease presentation and progression vary considerably according to age, ethnicity, and vascular phenotype, complicating early diagnosis and prognostic stratification [1,4,7,8].

Although MMD is most prevalent in East Asian countries such as Japan, Korea, and China, with reported incidence rates ranging from 0.34 to 0.94 per 100,000 inhabitants, the disease has been increasingly recognized worldwide, including in North America and Europe, albeit with lower incidence and distinct epidemiological patterns [5,6]. Female predominance and bimodal age distribution are well described in Asian cohorts but are less consistently observed in Western populations [1,7]. Historically, MMD was considered a diagnosis of exclusion. Advances in genetic research have identified susceptibility variants, particularly involving the RNF213 gene, yet genetic factors alone do not fully explain disease occurrence or progression, and environmental or acquired contributors appear to play a role [2,3]. As a result, imaging remains the cornerstone of diagnosis, disease staging, and follow-up.

Digital subtraction angiography (DSA) is widely accepted as the gold standard for diagnosing MMD, allowing detailed visualization of arterial stenosis, collateral networks, and disease severity using grading systems such as the Suzuki classification. However, DSA is invasive, involves ionizing radiation and contrast administration, and is not ideal for repeated assessment, particularly in pediatric patients; consequently, a wide array of noninvasive and minimally invasive imaging modalities has been developed to complement or partially substitute angiography, providing structural, hemodynamic, and functional information [3,9,10,11,12]. Therefore, given the heterogeneity of available imaging techniques and the lack of standardized comparative frameworks, this scoping review aims to comprehensively map the current imaging-based diagnostic approaches used in Moyamoya disease, summarize their reported diagnostic performance, and identify gaps that limit evidence synthesis and clinical translation.

## 2. Methods

### 2.1. Protocol and Reporting Standards

This scoping review was conducted in accordance with the Preferred Reporting Items for Systematic Reviews and Meta-Analyses extension for Scoping Reviews (PRISMA-ScR) guidelines (See the PRISMA-ScR checklist in the Table S1). The protocol was not registered in PROSPERO because at the time of development PROSPERO rejected the registration of any scoping review.

### 2.2. Eligibility Criteria

Eligible studies were original research articles published in English or Spanish that evaluated imaging modalities used for diagnosis, disease assessment, or functional characterization of Moyamoya disease or Moyamoya syndrome in human subjects. Observational study designs including cohort, case—control, cross-sectional, and retrospective studies were eligible. Studies were excluded if they were animal experiments, narrative reviews without original data, editorials, letters, expert opinions, or if they focused exclusively on surgical techniques without diagnostic imaging evaluation.

### 2.3. Information Sources and Search Strategy

A comprehensive literature search was conducted in PubMed, Scopus, and the Virtual Health Library from inception until May 2024. The search strategy combined controlled vocabulary and free-text terms related to Moyamoya disease and diagnostic imaging, including variants such as “Moyamoya syndrome,” “progressive intracranial occlusive arteriopathy,” and modality-specific terms such as “magnetic resonance imaging”, “computed tomography”, “multimodal imaging”, “angiography”, and “ultrasound”. The search strategy included the term “Moyamoya syndrome” to maximize sensitivity and ensure comprehensive retrieval of imaging studies, as terminology is inconsistently applied across the literature. However, given that Moyamoya syndrome comprises a heterogeneous group of conditions associated with distinct underlying etiologies, we only included studies looking specifically at Moyamoya disease and not Moyamoya syndrome.

### 2.4. Study Selection

Two blinded reviewers independently screened titles and abstracts using Zotero and Rayyan. Duplicates were removed by the reviewers and full-text screening was subsequently performed, and discrepancies were resolved by consensus and discussion with a third reviewer.

### 2.5. Data Extraction and Synthesis

Extracted variables included publication year, country, study design, sample size, patient demographics, imaging modality, reference standard, and reported diagnostic performance metrics including sensitivity, specificity, predictive values, and likelihood ratios when available. Due to heterogeneity in reporting, findings were synthesized narratively and organized by imaging modality.

## 3. Results

### 3.1. Study Selection and General Characteristics

**Figure 1:**
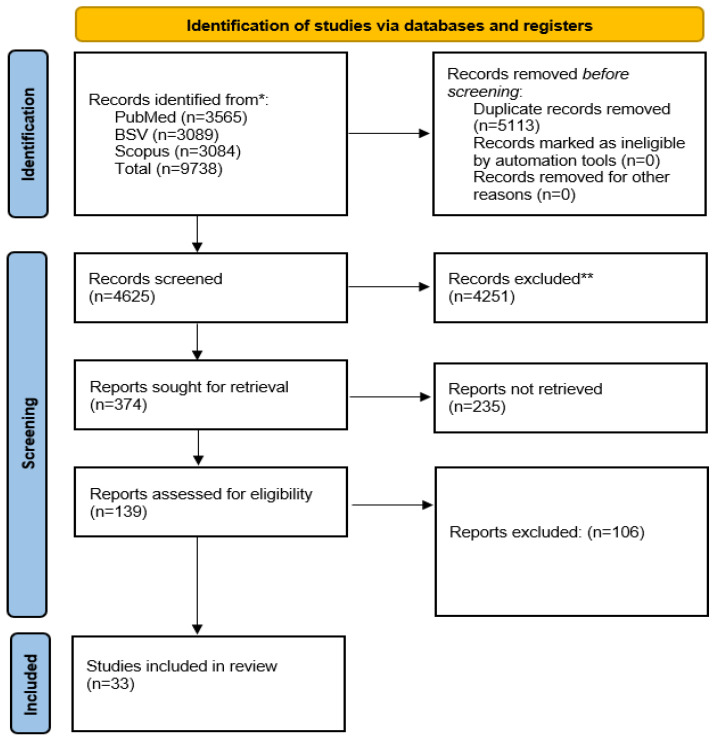
PRISMA Flow Diagram showcasing the study selection process. * scientific online repositories. ** manually with the aid of the web-based software Rayyan.

The systematic search retrieved 9738 records across PubMed, Scopus, and the Virtual Health Library. After removal of duplicates, 4625 records were screened by title and abstract. Following full-text assessment, 33 studies met the predefined eligibility criteria and were included in this scoping review. The study selection process is illustrated in the PRISMA flow diagram (Figure 1).

Included studies were published between 1995 and 2023 and originated predominantly from East Asia, particularly Japan, China, and South Korea, with additional studies from North America and Europe. Study designs were heterogeneous and included case—control, cross-sectional, cohort, and retrospective observational studies. Sample sizes ranged from single-case descriptions to large cohorts exceeding 700 participants. Digital subtraction angiography was employed as a reference standard in 30 of the 33 studies and was explicitly designated as the gold standard in 22 studies, primarily due to its superior spatial and temporal resolution, which allows detailed visualization of arterial stenosis, collateral vessel networks, and dynamic cerebral circulation, as well as its established role in disease staging using classifications such as the Suzuki grading system and in guiding surgical decision-making. A summary of the imaging modalities with established or potential uses in MMD can be found in Table 1.

### 3.2. Magnetic Resonance Imaging

From thirty-five articles, twenty-one mention magnetic resonance imaging (MRI) in different modalities, with a total sample of 668 patients with MMD with ages ranging from 1 to 68 years [9,11,12,13,14,15,16,17,18,19,20,21,22,23,24,25,26,27,28,29,30].

#### 3.2.1. Structural MRI and Brain Morphometry

Several studies employed high-resolution structural MRI to characterize parenchymal changes associated with Moyamoya disease. Volumetric analyses consistently demonstrated significant reductions in subcortical gray matter structures, including the thalamus, caudate nucleus, putamen, hippocampus, amygdala, pallidum, and nucleus accumbens [13]. These volumetric reductions showed significant negative correlations with disease duration, suggesting progressive neurodegeneration or chronic hypoperfusion-related injury [13]. Importantly, more than three-quarters of patients in these cohorts exhibited motor deficits, which were associated with structural and functional abnormalities in the primary motor cortex and its subcortical connections [13]. These findings support the concept that Moyamoya disease is not purely a vascular disorder but also involves secondary neurodegenerative processes. MR angiography (MRA) and structural MRI are highly effective in diagnosing MMD; in a prospective study including 26 patients with MMD aged 6 months to 54 years (mean age 14 years), they found a sensitivity of 73% for MR angiography and of 92% for structural MR, and a specificity of 100% for both modalities [29,30]. When combined, their diagnostic accuracy remains at 92% sensitivity and 100% specificity, providing a comprehensive assessment of the disease [29]. Additionally, brain MRI and MRA performed at symptom onset in childhood MMD, in a cohort of 26 pediatric patients aged 1 year 10 months–16 years 7 months (mean age 7.5 years), revealed characteristic findings that vary across developmental stages, highlighting the importance of early and stage-specific imaging evaluation [30].

#### 3.2.2. High-Resolution Vessel Wall MRI and Contras Enhanced MRI

Contrast-enhanced high-resolution MRI of the arterial wall (CE-HR-MRI) has been shown to provide detailed insights into disease activity and progression. Multiple studies demonstrated a characteristic concentric arterial wall enhancement pattern in affected vessels, reflecting active angiopathy rather than atherosclerotic plaque formation [13,14,15]. This enhancement pattern followed a reproducible temporal trajectory, persisting for approximately 12 months and subsequently diminishing, corresponding to disease stabilization [15]. CE-HR-MRI demonstrated sensitivity of 0.85 and specificity of 0.86 for predicting new ischemic symptoms within six months, with a particularly high negative predictive value of 0.95 [15]. Additional CE-HR-MRI studies identified specific vascular morphologies associated with hemorrhagic risk. Shortened total length of lenticulostriate arteries combined with preserved terminal internal carotid artery lumen was strongly associated with anterior intracerebral hemorrhage, achieving an area under the curve of 0.901 [14]. These findings highlight the prognostic value of vessel wall imaging beyond simple luminal assessment.

#### 3.2.3. MRI and MRA Field Strength Comparisons

Several studies compared the diagnostic performance of 3.0 T and 7.0 T MRI/MRA. Deng et al. reported superior sensitivity of 7.0 T MRI/MRA for diagnosing MMD based on time-of-flight criteria (TOF), with sensitivity of 1.000 and specificity of 0.933, compared with sensitivity of 0.692 for 3.0 T imaging [16]. Receiver operating characteristic analysis showed an area under the curve (AUC) of 0.851 (95% CI, 0.666–0.956) for 3.0 TMRI/MRA and 1.000 (95% CI 0.877–1.000) for 7.0T MRI/MRA according to TOF criteria [16]. The improved performance was primarily attributed to enhanced visualization of Moyamoya vessels and small collateral branches [16]. On the other hand, however, Oh et al., in a case—control study including 12 patients with MMD, aged 10–66 years (median age of 36 years), reported no significant differences between 3.0 T and 7.0 T MRI/MRA in Suzuki stage classification, internal carotid artery diameter, or ivy sign score [17]. Nonetheless, 7.0 T imaging demonstrated higher detection rates of flow voids on T2-weighted and TOF source images, suggesting improved collateral visualization; receiver operating characteristic analysis showed an AUC of 0.729 (95% CI, 0.583–0.862) for 3.0 T MRI and 0.931 (95% CI, 0.849–1.00) for 7.0T MRI according to T2 criteria, and 0.819 (95% CI. 0.694–0.945) versus 0.948 (95% CI 0.877–1.00) according to TOF criteria [17]. These findings indicate that ultra-high-field MRI could improve detection of small collateral vessels but does not uniformly enhance staging accuracy, underscoring the complexity of translating higher spatial resolution into clinical benefit and highlighting the need for more studies using high field equipment and standardized methodology.

Other MRA techniques included silent-MRA that emerged as a promising alternative to conventional TOF-MRA. While both techniques showed comparable performance in assessing steno-occlusive severity of major cerebral arteries, silent-MRA demonstrated significantly higher grading scores for Moyamoya vessels [18]. This improved visualization of collateral networks may enhance disease assessment while maintaining noninvasive acquisition, which is especially useful in pediatric patients. Dynamic susceptibility contrast MRI has also been proven useful by demonstrating that mean transit time correlates strongly with cerebrovascular reserve, particularly in anterior and middle cerebral artery territories in a study including 33 patients with MMD aged 18–64 years (mean age 38.3 years) [24]. Threshold values achieved a sensitivity of 68.4% and specificity of approximately 91% for predicting impaired cerebrovascular reserve, supporting its role as a functional assessment tool [24].

#### 3.2.4. Arterial Spin Labeling MRI

Arterial spin labeling (ASL) MRI was extensively evaluated as a noninvasive method for assessing cerebral perfusion and hemodynamic compromise. Hara et al. demonstrated that visual scoring of ASL images correlated strongly with PET-derived oxygen extraction fraction, enabling estimation of misery perfusion without radiation or contrast agents; the use of dual post-labeling delays improved assessment accuracy and allowed gross evaluation of cerebrovascular reserve in routine clinical settings [19]. Donahue et al. further showed that ASL and BOLD imaging provided critical information regarding infarct risk that was not available from structural MRI or DSA alone [20]. These findings position ASL as a complementary modality rather than a replacement for angiography. Furthermore, super-selective pseudo-continuous ASL has also demonstrated high sensitivity and specificity for delineating external carotid artery perfusion territories, with diagnostic performance comparable to DSA, in a prospective study including 103 patients with MMD (mean age 39.6 years, range 19–60 years), showing moderate intermodality agreement (κ = 0.49; 95% CI, 0.43–0.55) and good inter-reader agreement (κ = 0.71; 95% CI, 0.66–0.76) [21]. This technique may reduce the need for repeated invasive angiography during follow-up and eliminates the need for contrast administration; this is relevant for specific patient populations, such as those with allergies or renal conditions.

#### 3.2.5. Susceptibility-Weighted Imaging

Susceptibility-weighted imaging (SWI) with minimum intensity projection has provided valuable information on venous architecture and iron deposition; asymmetric venous vascularity on SWI has been shown to correlate with higher Suzuki stages and advanced stenosis [22]. SWI could have a diagnostic sensitivity comparable to MRA and electroencephalography. Certainly, high-intensity SWI has been used to identify dilation and extension of the anterior choroidal and posterior communicating arteries as independent risk factors for cerebral microbleeds, which were predictive of subsequent intraventricular hemorrhage [25]. These findings highlight the role of venous and microhemorrhagic markers in hemorrhagic risk stratification of MMD patients.

#### 3.2.6. Microstructural and Myelin-Sensitive MRI

Myelin-sensitive MRI and diffusion imaging can reveal widespread white matter abnormalities in MMD. Significant reductions in myelin and axon volume fractions have been observed across multiple white matter regions [23]. Importantly, axonal damage correlates more strongly with cognitive impairment than myelin loss, suggesting that chronic hypoperfusion may preferentially disrupt axonal integrity in MMD patients [23].

### 3.3. Computed Tomography-Based Imaging

#### 3.3.1. CT Perfusion and Source Images

Computed tomography perfusion source images (CTP-Sis) offer a noninvasive method for assessing collateral flow in patients with unilateral MMD. In a study including 24 patients with unilateral MMD (mean age 30.7–10.1 years; range 14–50 years), CTP-Sis demonstrated a sensitivity of 0.714 (95% CI, 0.578–0.851) and a specificity of 0.995 (95% CI, 0.985–1.000) [31]. Notably, CTP-Sis readers showed greater consistency in scoring compared to digital subtraction angiography (DSA) readers, highlighting its potential as a reliable alternative for collateral evaluation in clinical practice [31].

Additionally, in the evaluation of cerebrovascular reserve (CVR) in MMD, computed tomography perfusion (CTP) has demonstrated significant potential, particularly when combined with an acetazolamide challenge and appropriate normalization techniques [32]. While baseline CTP parameters such as cerebral blood volume (CBV) and mean transit time (MTT) showed only weak correlations with SPECT-derived CVR, the percent change in cerebral blood flow (pcCBF) exhibited a strong and consistent correlation across all vascular territories [32]. This robust association positions pcCBF as a reliable surrogate marker for cerebrovascular reserve. Given the wider accessibility and lower cost of CTP compared to SPECT, it may serve as a practical and effective noninvasive alternative for CVR assessment in clinical practice [32].

#### 3.3.2. Xenon-Enhanced CT

Xenon-enhanced computed tomography (XeCT) has provided valuable insights into cerebral hemodynamics in MMD, revealing a distinctive perfusion pattern characterized by relative central preservation of cerebral blood flow (CBF) [33]. Although both cortical and central CBF significantly decline with age, cerebrovascular reserve capacity (CVRC) and hemodynamic stress distribution (hdSD) remain relatively stable, suggesting that these parameters may serve as more reliable indicators of disease burden [33]. In patients with MMD, baseline CBF and CVRC in cortical territories have been shown to be markedly reduced compared to controls, while hdSD, particularly under stimulation, is significantly elevated [33]. These findings support the use of CVRC and hdSD as potentially useful markers of hemodynamic compromise in MMD, offering region-specific information that may exceed the diagnostic utility of CBF measurements alone [33].

#### 3.3.3. CT Angiography

Computed tomography angiography (CTA), particularly when performed with multidetector row computed tomography (MDCT), has emerged as a reliable and practical alternative to magnetic resonance angiography (MRA) for evaluating steno-occlusive changes in MMD [34]. In a study including 24 patients with MMD or unilateral MMD (mean age 30.7–16.5 years; range 3–60 years), CTA demonstrated a significantly higher rate of detection of Moyamoya-affected vessels and showed strong correlation with MRA scores, while also providing greater sensitivity in identifying mild stenosis and small distal vessels [34]. Unlike MRA, which may overestimate stenosis severity in some cases, CTA offers superior spatial resolution that enables more accurate assessment of vascular lesions [34]. The rapid acquisition and ease of use without the need for specialized equipment make CTA especially valuable in pediatric populations, emergency settings, or resource-limited situations. These findings support the use of CTA with MDCT as a dependable and efficient tool for the diagnosis and monitoring of MMD, with diagnostic performance that is not inferior to conventional angiography [34].

The main reported diagnostic accuracy of selected imaging techniques used in MMD are shown in Table 2.

### 3.4. Nuclear Medicine Techniques

Oxygen extraction fraction (OEF), relative cerebral blood flow (RCBF), and relative cerebrovascular reactivity (RCVR) to acetazolamide, assessed via brain perfusion single-photon emission computed tomography (SPECT), can offer key insights into ischemic pathology in MMD [35]. Studies have revealed that RCBF had superior diagnostic performance compared to RCVR and RCBF, showing significantly greater specificity and positive predictive value [35]. Moreover, combining RCBF and RCVR did not enhance predictive accuracy beyond RCBF alone; correlation analysis indicated a strong association between OEF and RCBF and a weaker inverse correlation with RCVR, underscoring the higher reliability of RCBF in identifying hemodynamic compromise in ischemic MMD [35]. In another study, the presence or distribution of cortical microvascularization (CM) did not significantly differ according to regional cerebrovascular reserve (rCVR) status in hemispheres with reduced middle cerebral artery (MCA) flow [36]. Interestingly, in hemispheres with preserved MCA flow, CM was more frequently observed in regions with decreased perfusion compared to those with normal perfusion, suggesting that CM may represent an early compensatory mechanism [36]. Furthermore, acetazolamide stress SPECT studies indicate that CM may be detectable even at early stages of MMD, when antegrade flow and rCVR remain intact, highlighting its potential as a marker of early microvascular adaptation before overt hemodynamic compromise occurs [36].

### 3.5. Ultrasound and Neurophysiological Techniques

Transcranial Doppler ultrasound has demonstrated characteristic flow velocity and pulsatility index patterns corresponding to MMD angiographic stages [37]. In a cohort of 90 patients with MMD confirmed by digital subtraction angiography (mean age 32.49–13.02 years), mean flow velocity of the terminal internal carotid artery, measured through color duplex ultrasound, achieved an AUC of 0.776 (95% CI 0.657–0.894) for detecting >50% stenosis and 0.734 (95% CI 0.647–0.821) for detecting occlusion [38]. In a cross sectional analytical observational study including 45 patients, the value of transcranial Doppler ultrasound as a non-invasive method for estimating the hemodynamic severity of MMD was assessed, using magnetic resonance angiography as the primary comparator and digital subtraction angiography as the reference standard in a subpopulation in a cohort of 37 women and 8 men, with a mean age of 34.9 ± 11.4 years [37]. Based on magnetic resonance angiography, cerebral hemispheres were classified into three stages according to the degree of arterial stenosis. Stage 1, corresponding to mild stenosis, is characterized by relatively preserved arterial lumen with normal or mildly increased mean flow velocities and preserved pulsatility indices. Stage 2, corresponding to moderate stenosis, is associated with elevated mean flow velocities greater than 85 cm/s and reduced pulsatility indices below 0.60, reflecting compensatory hemodynamic changes. Stage 3, representing severe stenosis or near occlusion, is characterized by low mean flow velocities below 50 cm/s and increased pulsatility indices, indicating advanced hemodynamic compromise [37]. These parameters demonstrated good discriminative capacity between stages; among the 34 patients who also underwent digital subtraction angiography, magnetic resonance angiography-based classification showed a 93% concordance rate, supporting its validity as a reference method [37]. Overall, these findings indicate that transcranial Doppler can reliably reflect the degree of stenosis defined by magnetic resonance angiography and digital subtraction angiography, supporting its potential role as an accessible and non-invasive tool for functional assessment and longitudinal monitoring [37]. Furthermore, in a separate cross sectional analytical observational study including 90 patients, the utility of color Doppler ultrasound, incorporating transcranial color coded sonography and cervical color Doppler ultrasound, was evaluated for the non-invasive detection of terminal internal carotid artery steno-occlusion; the study population was stratified into three groups according to digital subtraction angiography findings, which served as the reference standard, including terminal internal carotid artery occlusion, stenosis greater than 50%, and stenosis of 50% or less [38]. Analysis of mean flow velocity enabled the identification of diagnostic cut off values; for stenosis greater than 50%, a mean flow velocity above 88.5 cm/s achieved an area under the curve of 0.776, with a sensitivity of 62.5% and a specificity of 88.15% [38]. For occlusion, a mean flow velocity below 49.5 cm/s yielded an area under the curve of 0.734, with a sensitivity of 55.56% and a specificity of 83.81% [38]. In addition, color Doppler ultrasound detected an absence of flow signal in 12 terminal internal carotid arteries, all of which were confirmed as complete occlusions by digital subtraction angiography [38]. These results support the potential use of mean flow velocity measured by color Doppler ultrasound as a practical and widely available functional parameter for estimating stenosis severity in MMD [38].

Finally, pattern-reversal visual-evoked potentials (P-VEP) have emerged as a practical and cost-effective tool for assessing posterior cerebral artery (PCA) occlusion in patients with MMD [39]. In comparative analyses, abnormalities detected through P-VEP were found to be highly specific to PCA involvement; although positron emission tomography (PET) remains the most reliable modality for confirming PCA compromise, its limited availability and high cost hinder routine clinical application [39]. Therefore, P-VEP represents a potential valuable, noninvasive alternative for the early detection of PCA involvement in the course of MMD progression [39].

### 3.6. Digital Subtraction Angiography and Artificial Intelligence

DSA remained the reference standard in the majority of studies. Angiographic markers including leptomeningeal collaterals, anterior choroidal artery dilation, and posterior communicating artery to internal carotid artery ratio correlated with disease severity and hemorrhagic risk, with ethnic variability were noted [40,41]. In an angiographic study, including Suzuki stage 3 pediatric MMD participants, a progressive reduction in the median grade of antegrade middle cerebral artery (MCA) flow was noted as MMD severity increased [36]. Furthermore, cortical microvascularization (CM), particularly in anterior regions, was frequently detected in hemispheres during the early stages of the disease, but its prevalence decreased as the condition progressed; the simultaneous presence of basal collaterals (BCs) and early CM, even in hemispheres with preserved antegrade MCA flow and normal regional cerebrovascular reserve (rCVR), suggests that CM may serve as an early angiographic indicator of microvascular remodeling in MMD [36]. Additionally, angiographic evaluation of MMD has shown that dilatation and abnormal collateral branching of the anterior choroidal artery (AChA) and posterior communicating artery (P-CoM) are strongly associated with hemorrhagic events, as shown in a study including 107 patients with angiographically confirmed MMD, comprising 70 patients with ischemic presentation and 37 with hemorrhagic presentation, stratified into 47 patients aged ≤20 years and 60 patients aged >20 years [40]. Grade 2 changes in the AChA have been observed in the majority of hemorrhagic hemispheres, while P-CoM alterations, though less frequent, remained significantly more common in hemorrhagic than in ischemic or asymptomatic hemispheres [40]. The combined presence of dilated AChA and/or P-CoM achieved a sensitivity of 84.4% and specificity of 86.4% for predicting hemorrhagic events, with a positive predictive value of 69.2% and a negative predictive value of 93.8%; these findings highlight the prognostic utility of detailed angiographic assessment of these arteries in identifying patients at elevated risk for intracranial bleeding in MMD [40]. This is even more significant if deep learning is combined with DSA; studies have achieved diagnostic accuracies exceeding 97 percent, supporting automated analysis as an adjunct to expert interpretation [42].

## 4. Discussion

This scoping review provides a comprehensive synthesis of imaging-based diagnostic approaches in MMD, highlighting the progressive transition from purely structural angiographic evaluation toward a multimodal framework integrating anatomical, hemodynamic, microstructural, and functional information. The reviewed literature demonstrates that while digital subtraction angiography remains indispensable for definitive diagnosis and surgical planning, no single imaging modality fully captures the complexity of MMD’s pathophysiology. Instead, complementary techniques are required to address the heterogeneous clinical phenotypes, age-dependent manifestations, and variable risks of ischemic and hemorrhagic events.

Digital subtraction angiography continues to be the cornerstone of diagnosis and classification. Its unparalleled spatial and temporal resolution allows precise visualization of arterial stenosis, collateral networks, and disease staging using the Suzuki classification. Across the included studies, DSA was used as the reference standard in the vast majority of diagnostic comparisons and remains essential for confirming diagnosis, determining surgical candidacy, and guiding revascularization strategies [40,41]. Importantly, angiographic analyses have moved beyond luminal stenosis alone. Several studies demonstrated that specific collateral patterns, including leptomeningeal collaterals, dilation and abnormal branching of the anterior choroidal artery, and increased posterior communicating artery to internal carotid artery ratios, correlate with disease severity and hemorrhagic risk [40,41]. However, these associations appear to vary by ethnicity, as anterior choroidal artery changes were less specific for hemorrhage in North American cohorts compared with Asian populations, underscoring the need for population-specific risk stratification models [41]. Despite its strengths, DSA is limited by invasiveness, radiation exposure, contrast administration, and reduced feasibility for serial monitoring, particularly in pediatric patients. These limitations have driven the development and adoption of noninvasive imaging techniques that can complement angiographic findings and provide additional functional insight.

On the other hand, magnetic resonance imaging has emerged as the most versatile and informative noninvasive modality in MMD, offering structural, vascular, perfusion, and microstructural evaluation within a single examination. The reviewed studies collectively demonstrate that MRI contributes critical information beyond luminal stenosis, particularly regarding disease activity, hemodynamic compromise, and secondary parenchymal injury [9,11,12,13,14,15,16,17,18,19,20,21,22,23,24,25,26,27,28,29,30]. Structural MRI and morphometric analyses consistently revealed subcortical volume reductions in patients, involving regions integral to motor, cognitive, and affective processing [13]. The correlation between volumetric loss and disease duration suggests a cumulative injury mechanism likely driven by chronic hypoperfusion, recurrent ischemic insults, and altered neurovascular coupling. These findings challenge the traditional view of MMD as a purely vascular disorder and support its conceptualization as a progressive neurovascular and neurodegenerative condition. Furthermore, high-resolution vessel wall MRI represents a major advance in understanding disease activity; the identification of concentric arterial wall enhancement patterns associated with active angiopathy provides a noninvasive marker of disease progression that is not accessible through conventional angiography [15]. The demonstrated sensitivity and specificity for predicting near-term ischemic events suggest a potential role for vessel wall MRI in risk stratification and treatment timing. Additionally, the association between specific vessel wall morphologies and hemorrhagic risk further emphasizes the prognostic value of this technique [14]. Ultra-high-field MRI has shown improved visualization of small collateral vessels and Moyamoya networks, particularly at 7.0 T [16]. However, the lack of consistent improvement in Suzuki staging or major arterial assessment across studies highlights an important distinction between image quality and clinical utility [17]. While higher field strength enhances spatial resolution, its incremental benefit may be greatest in research settings or complex cases rather than routine clinical practice. Finally, the emergence of Silent-MRA addresses some limitations of conventional TOF-MRA by improving collateral visualization without sacrificing noninvasiveness [18]. This technique may be particularly valuable for longitudinal follow-up, where repeated DSA is impractical. An important clinical consideration in the diagnostic evaluation of intracranial steno-occlusive vasculopathies is the differentiation of primary Moyamoya disease from secondary causes of moyamoya-like vascular patterns. Although the present review focused exclusively on Moyamoya disease to preserve etiological specificity, imaging modalities can be useful in supporting this distinction in clinical practice. High-resolution vessel wall MRI has emerged as a particularly valuable tool in this context. Moyamoya disease is typically characterized by concentric, homogeneous arterial wall thickening and enhancement, reflecting a non-atherosclerotic, non-inflammatory arteriopathy, whereas secondary vasculopathies such as atherosclerosis or vasculitis often demonstrate eccentric wall thickening, irregular enhancement patterns, and associated luminal features [13,14,15,16,17,18]. In addition, multimodal imaging approaches integrating structural, perfusion, and angiographic data may provide indirect clues to underlying etiology. The bilateral and progressive involvement of the terminal internal carotid arteries, together with the development of characteristic basal collateral networks, supports a diagnosis of Moyamoya disease, while asymmetric, focal, or atypical vascular patterns may suggest alternative causes [3,5]. Functional imaging techniques, including perfusion MRI and SPECT, may further contribute by identifying patterns of hemodynamic compromise consistent with chronic progressive arteriopathy rather than acute or segmental vascular insults [19,35]. Therefore, although definitive differentiation may require integration of clinical, genetic, and laboratory data, advanced imaging, particularly vessel wall MRI and multimodal strategies, provides useful noninvasive markers that can guide diagnostic reasoning and improve specificity in distinguishing Moyamoya disease from other intracranial vasculopathies.

Hemodynamic impairment is central to the clinical manifestations of MMD, yet conventional angiography provides limited functional information. Perfusion imaging techniques bridge this gap by directly assessing cerebral blood flow, cerebrovascular reserve, and oxygen extraction. Arterial spin labeling MRI stands out as a practical and increasingly validated tool. The strong correlation between ASL-derived perfusion patterns and PET-measured oxygen extraction fraction supports its use as a surrogate marker of misery perfusion [19]. Importantly, ASL requires neither contrast agents nor radiation, making it particularly suitable for pediatric populations and repeated monitoring. Vessel-selective ASL further enhances its utility by enabling assessment of collateral perfusion territories with diagnostic accuracy comparable to DSA [21]. Dynamic susceptibility contrast MRI provides quantitative hemodynamic metrics, particularly mean transit time, which correlate strongly with cerebrovascular reserve [24]. However, its reliance on contrast agents limits repeated use, and standardized thresholds for clinical decision-making remain underdeveloped.

Microstructural analysis has also shown to be beneficial, susceptibility-weighted imaging contributes unique information regarding venous architecture, iron deposition, and microhemorrhagic changes. The association between asymmetric venous vascularity and advanced disease stages suggests that venous alterations may reflect chronic hemodynamic stress and impaired oxygen extraction [22]. Furthermore, the identification of microbleeds and dilated collateral arteries as predictors of future hemorrhage highlights the potential of SWI for hemorrhagic risk stratification [25]. Microstructural MRI studies have revealed widespread white matter injury, with axonal damage more strongly associated with cognitive impairment than myelin loss [23]. These findings suggest that cognitive dysfunction in MMD may result not only from overt strokes but also from diffuse microstructural injury driven by chronic hypoperfusion. This has important implications for early diagnosis and intervention, particularly in asymptomatic or mildly symptomatic patients.

Computed tomography-based imaging remains highly relevant, particularly in acute settings, pediatric populations, and regions with limited access to advanced MRI. Multidetector CTA consistently demonstrated high sensitivity for detecting distal vessels and mild stenosis, often outperforming MRA [34]. Its rapid acquisition and high spatial resolution make it particularly useful in emergency contexts. CT perfusion imaging, especially when combined with acetazolamide challenge, provides accessible assessment of cerebrovascular reserve [32]. The strong correlation between percentage change in cerebral blood flow and SPECT-derived reserve underscores its functional relevance; however, radiation exposure and contrast use limit its suitability for frequent monitoring. Xenon-enhanced CT offers unique quantitative assessment of cerebral hemodynamics, including cerebrovascular reserve capacity and hemodynamic stress distribution [33]. Although its availability is limited, the robustness of these parameters suggests potential value in complex cases or research settings.

When looking at other techniques that are being studied, SPECT remains an important modality for assessing cerebral perfusion and reserve, particularly where PET is unavailable. Relative cerebral blood flow demonstrated superior diagnostic performance compared with cerebrovascular reactivity, supporting its use in ischemic risk assessment [35]. The observation that cortical microvascularization may represent an early compensatory mechanism highlights the potential of functional imaging for early disease detection [36]. Neurophysiological techniques such as pattern-reversal visual evoked potentials provide low-cost functional assessment of posterior circulation involvement [39]. While limited in scope, these techniques may be valuable in resource-limited settings or as adjunctive screening tools. Finally, ultrasound-based techniques, including transcranial Doppler and color duplex ultrasound, offer accessible bedside assessment of hemodynamic severity [37,38]. Their operator dependence and limited spatial resolution restrict their role, yet they remain useful for screening and follow-up.

To further contextualize the clinical utility of the reviewed imaging modalities, a comparative appraisal of their strengths, limitations, and optimal applications is essential. Digital subtraction angiography remains indispensable for definitive diagnosis and surgical planning due to its superior spatial and temporal resolution; however, its invasiveness, radiation exposure, and limited feasibility for serial monitoring restrict its role primarily to baseline confirmation and preoperative assessment [3,40,41]. In contrast, magnetic resonance-based techniques provide the most comprehensive noninvasive evaluation, integrating structural, hemodynamic, and microstructural information within a single examination [9,11,12]. Structural MRI and MRA are particularly suitable for initial evaluation and longitudinal follow-up, while vessel wall imaging offers unique insight into disease activity and short-term ischemic risk [13,14,15]. Perfusion techniques such as arterial spin labeling are especially advantageous in pediatric populations and for repeated assessments, given their noninvasive nature and absence of contrast or radiation, although they remain limited by lower spatial resolution and variability in acquisition protocols [19,20,21]. On the other hand, computed tomography-based modalities occupy an important role in acute and resource-limited settings; CT angiography provides rapid and widely accessible assessment of vascular anatomy with high sensitivity for distal vessel involvement, making it particularly useful in emergency evaluation or when MRI is contraindicated [34]. CT perfusion, especially when combined with vasoactive challenge, offers useful information regarding cerebrovascular reserve; however, radiation exposure and contrast use limit its application in longitudinal monitoring and studies [31,32]. Nuclear medicine techniques such as SPECT remain valuable for assessing hemodynamic compromise and cerebrovascular reserve, particularly in centers without access to advanced MRI or PET, although their spatial resolution and availability are limited [35,36]. Finally, ultrasound-based methods, including transcranial Doppler and color duplex sonography, provide low-cost, bedside evaluation of hemodynamic severity and are well suited for screening and follow-up, but their operator dependence and limited anatomical detail restrict their diagnostic role to those centers with highly specialized personnel [37,38].

**Figure 2:**
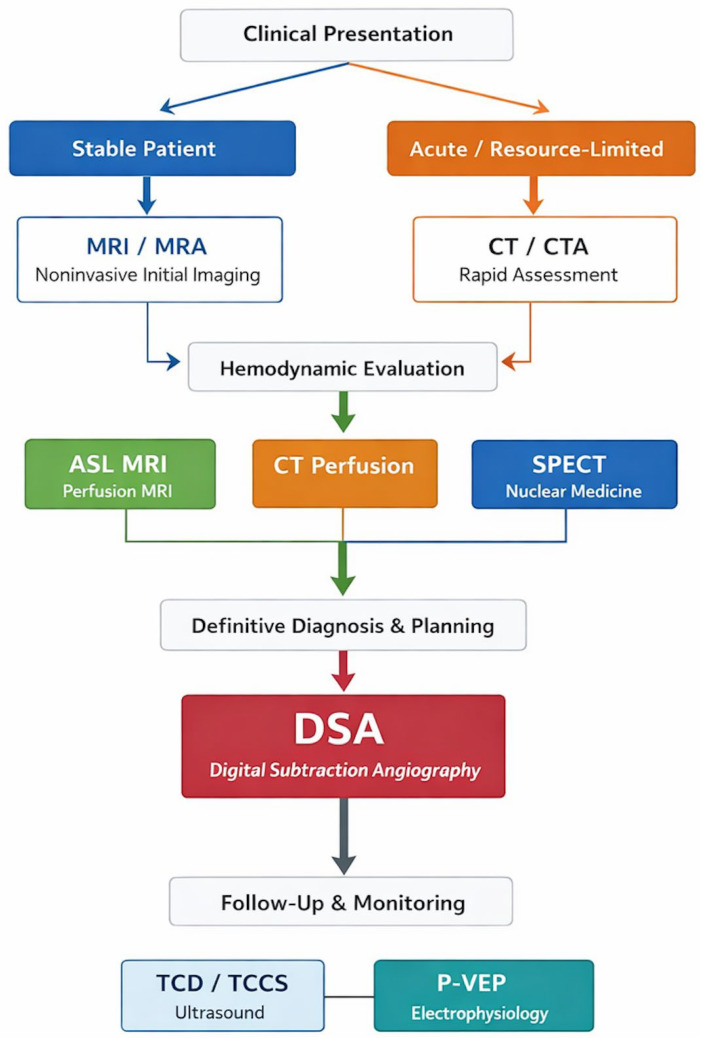
Suggested Multimodal Imaging Workflow in Moyamoya Disease. Proposed stepwise imaging approach for Moyamoya disease integrating clinical presentation and resource availability. Initial evaluation typically involves noninvasive imaging with MRI/MRA for structural and vascular assessment. In acute or resource-limited settings, CT/CTA may serve as an alternative. Hemodynamic assessment is performed using perfusion techniques such as ASL MRI, CT perfusion, or SPECT, depending on availability. Digital subtraction angiography is reserved for definitive diagnosis, staging, and surgical planning. Ultrasound-based techniques may support follow-up and longitudinal monitoring. This multimodal framework emphasizes tailored imaging selection based on patient characteristics and clinical context [3,19,31,34,35,37,40].

Importantly, no single modality is sufficient to fully characterize the complex pathophysiology of Moyamoya disease; instead, imaging selection should be tailored to the clinical context. Noninvasive MRI-based approaches are most appropriate for screening, longitudinal monitoring, and evaluation of disease activity; CT-based techniques are advantageous in acute or resource-limited settings; and DSA remains essential for definitive diagnosis and surgical decision-making [3,19,31,34,40]. A multimodal imaging strategy, guided by patient age, clinical presentation, resource availability, and expertise is therefore critical for optimizing diagnostic accuracy and clinical management [1,2,3]. We propose a multimodal imaging workflow in Figure 2.

### 4.1. Age-Related Differences in Imaging Strategies: Pediatric Versus Adult Moyamoya Disease

Pediatric and adult Moyamoya disease exhibit distinct clinical phenotypes, progression patterns, and imaging priorities, necessitating age-specific diagnostic approaches [43]. Pediatric patients more frequently present with ischemic symptoms, including transient ischemic attacks and infarctions, whereas adults more commonly present with hemorrhagic events, particularly intracerebral or intraventricular hemorrhage [1,3,7]. These differences have important implications for imaging selection and interpretation. In pediatric populations, imaging strategies should prioritize early detection of hemodynamic compromise and minimization of radiation exposure; therefore, noninvasive modalities such as MRI/MRA and arterial spin labeling could be particularly advantageous, as they allow repeated assessment of cerebral perfusion and cerebrovascular reserve without the need for contrast agents or ionizing radiation [19,20,21]. Additionally, early-stage imaging may reveal subtle perfusion abnormalities or collateral formation before infarction, supporting timely intervention. Techniques such as Silent-MRA and vessel-selective ASL are especially valuable in this context, given their ability to characterize collateral circulation noninvasively [18,21]. In contrast, adult patients often require imaging approaches tailored to hemorrhagic risk stratification and evaluation of complex collateral networks. Susceptibility-weighted imaging is particularly useful in these patients for detecting cerebral microbleeds and venous abnormalities associated with increased hemorrhagic risk [22,25]. High-resolution vessel wall MRI also provides important insights into vascular remodeling and may assist in identifying imaging features associated with future ischemic or hemorrhagic events [14,15]. Furthermore, angiographic assessment, including digital subtraction angiography, remains particularly relevant in adults for detailed evaluation of collateral patterns and surgical planning [40,41].

Across both populations, multimodal imaging remains essential. Pediatric imaging strategies prioritize longitudinal monitoring and radiation-free techniques, while adult imaging more frequently focuses on risk stratification for hemorrhage and advanced vascular characterization. Recognizing these age-dependent differences is important for optimizing diagnostic pathways and tailoring imaging protocols to individual patient needs.

### 4.2. Future Directions

The evolving landscape of imaging in MMD underscores the need for coordinated, methodologically robust research efforts aimed at improving diagnostic accuracy, prognostic stratification, and clinical decision-making. Future investigations should move beyond single-modality descriptions toward integrated, standardized, and outcome-oriented imaging frameworks (i.e., multimodality imaging). A major priority should be the standardization of imaging protocols and reporting. Across the reviewed studies, substantial heterogeneity exists in acquisition parameters, post-processing techniques, outcome definitions, and reference standards. This variability severely limits cross-study comparison and precludes large-scale meta-analytic synthesis. Future studies should adopt harmonized reporting frameworks for diagnostic accuracy, including mandatory reporting of true positives, false positives, true negatives, and false negatives whenever feasible. Such standardization would enable pooled analyses and more precise estimation of modality-specific performance.

Another critical direction involves the integration of multimodal imaging; MMD is fundamentally a neurovascular disorder with intertwined structural, hemodynamic, and microstructural components [1,2,3,4,5,6,7,8]. Future research should prioritize prospective studies that combine angiographic, perfusion, vessel wall, susceptibility, and microstructural imaging within the same patient cohorts. Multimodal imaging may allow identification of composite biomarkers that better reflect disease activity, ischemic vulnerability, and hemorrhagic risk than any single modality alone. Longitudinal imaging studies in Moyamoya disease remain limited in number and, more importantly, in methodological consistency, despite the emergence of several recent investigations employing longitudinal designs. Existing studies have provided valuable insights into the temporal evolution of perfusion deficits, collateral development, and microstructural changes; however, most are characterized by relatively small sample sizes, single-center cohorts, heterogeneous imaging protocols, and variable follow-up intervals, which restrict generalizability and limit cross-study comparability. These limitations underscore that the current evidence base remains insufficient to define standardized imaging biomarkers of disease progression or treatment response. Several practical challenges contribute to this limitation such as the fact that long-term follow-up in MMD is inherently complex due to variability in disease progression, which may range from rapid deterioration to prolonged stability, particularly following revascularization. In addition, differences in clinical presentation between pediatric and adult populations, as well as variability in access to specialized imaging, further complicate the design of longitudinal cohorts. Although life expectancy in treated patients with MMD has improved substantially and may approach that of the general population in some cohorts, untreated or progressive disease remains associated with a significant risk of recurrent ischemic or hemorrhagic events, which may impact long-term retention and follow-up feasibility [3,5].

The role of functional and hemodynamic imaging warrants further refinement. Techniques such as arterial spin labeling MRI, dynamic susceptibility contrast MRI, CT perfusion with acetazolamide challenge, and SPECT have demonstrated promise in assessing cerebrovascular reserve and misery perfusion. However, clinically actionable thresholds remain poorly defined. Future work should aim to establish validated cut-off values linked to stroke risk, cognitive decline, or hemorrhagic events, thereby translating functional imaging findings into concrete clinical decisions.

Pediatric-specific imaging strategies represent another crucial area for development. Children with Moyamoya disease often present with different clinical phenotypes and face unique risks related to radiation exposure and repeated invasive procedures. Research efforts should focus on optimizing radiation-free modalities, such as ASL MRI and Silent-MRA, and on defining age-specific imaging biomarkers that capture early disease activity before irreversible injury occurs.

The expanding application of artificial intelligence and machine learning in Moyamoya imaging offers substantial opportunities [42,44]. While early studies applying deep learning to DSA interpretation have demonstrated high diagnostic accuracy [42], future research should extend beyond detection toward prognostication and treatment planning. Integrating AI models with multimodal imaging data may enable automated risk stratification, prediction of ischemic or hemorrhagic events, and individualized follow-up strategies. Importantly, such models must be externally validated across diverse populations to ensure generalizability. Certainly, ethnic and geographic variability observed in angiographic predictors of disease severity and hemorrhagic risk highlights the need for population-specific validation studies. Imaging biomarkers derived from East Asian cohorts may not directly translate to Western populations. Future multicenter and multinational studies are required to clarify how genetic background, vascular anatomy, and collateral patterns influence imaging interpretation and clinical risk.

Finally, future longitudinal studies should prioritize standardized, multimodal imaging protocols integrating angiographic, perfusion, vessel wall, and microstructural techniques within the same patient cohorts. Optimal study design should include clearly defined baseline imaging at diagnosis, short-term follow-up to assess disease activity and early treatment response (e.g., within 3–6 months), and longer-term intervals to evaluate progression and clinical outcomes (e.g., annually). Importantly, imaging findings should be systematically linked to clinically meaningful endpoints, including stroke recurrence, cognitive decline, and hemorrhagic events. Multicenter collaboration and harmonized acquisition protocols will be essential to overcome current limitations and enable robust identification of prognostic imaging biomarkers.

### 4.3. Methodological Limitations and Evidence Gaps

Despite the expanding imaging literature on MMD, several methodological limitations and evidence gaps continue to restrict the interpretability and clinical translation of current findings. The reviewed studies exhibit substantial heterogeneity in design, ranging from small retrospective cohorts to cross-sectional case—control analyses, often with limited sample sizes and inconsistent inclusion criteria. Definitions of Moyamoya disease and Moyamoya syndrome were not uniformly applied, and patient populations varied widely with respect to age, disease stage, and clinical presentation, introducing selection bias and limiting cross-study comparability. A further limitation relates to variability in reference standards and outcome reporting. Although digital subtraction angiography is widely regarded as the diagnostic gold standard, its use was not uniform, and some studies relied on MRI or CT-based modalities as surrogate references. In addition, angiographic grading systems were inconsistently applied. Diagnostic accuracy metrics were incompletely reported in most studies, with only a minority providing sufficient data to derive sensitivity, specificity, or predictive values. This lack of standardized reporting directly precluded robust meta-analytic synthesis and underscores the need for harmonized diagnostic accuracy frameworks. Furthermore, most available evidence is cross-sectional, with a marked scarcity of longitudinal imaging studies; as a result, the temporal evolution of imaging biomarkers, their stability over time, and their relationship with clinical outcomes remain poorly defined. Few studies directly compared multiple imaging modalities within the same cohort using standardized protocols, limiting comparative effectiveness assessment and obscuring the potential synergistic value of multimodal imaging approaches.

Additional gaps include underrepresentation of pediatric populations in advanced imaging studies, limited evaluation of ethnic and geographic variability in imaging predictors, and the early developmental stage of artificial intelligence applications, which remain largely unvalidated outside single-center settings. Collectively, these limitations highlight the need for standardized, longitudinal, and multimodal imaging studies across diverse populations to strengthen the evidence base and enable translation into clinically actionable diagnostic and prognostic strategies. Future research should prioritize standardized diagnostic accuracy frameworks, consistent reference standards, and longitudinal designs that link imaging findings with clinical outcomes. Such efforts are essential to translate the growing body of imaging research into evidence-based diagnostic algorithms.

## 5. Conclusions

This scoping review provides a comprehensive synthesis of imaging-based diagnostic approaches in Moyamoya disease, highlighting the central role of imaging in diagnosis, disease characterization, and clinical decision-making. Digital subtraction angiography remains the definitive reference standard for confirming diagnosis, staging disease severity, and guiding revascularization strategies. However, the evidence consistently demonstrates that angiography alone is insufficient to capture the full structural, hemodynamic, and neurobiological complexity of MMD. Noninvasive imaging modalities, particularly magnetic resonance-based techniques, have emerged as essential complements to angiography. High-resolution vessel wall imaging offers insight into disease activity and progression, perfusion techniques such as arterial spin labeling enable assessment of cerebrovascular reserve without radiation or contrast, and susceptibility and microstructural imaging reveal secondary parenchymal injury and hemorrhagic risk markers. Computed tomography-based approaches, nuclear medicine techniques, ultrasound, and neurophysiological methods provide valuable alternatives or adjuncts in specific clinical contexts, particularly when access, urgency, or patient characteristics limit MRI use.

Despite significant technological advances, the current literature is constrained by methodological heterogeneity, limited longitudinal data, incomplete diagnostic accuracy reporting, and inconsistent multimodal integration. These limitations preclude definitive comparative effectiveness conclusions and restrict translation into standardized diagnostic algorithms. Future progress will depend on harmonized imaging protocols, longitudinal and multimodal study designs, population-diverse cohorts, and clinically oriented outcome validation.

## Figures and Tables

**Table 1:** Summary of Imaging Modalities Used in Moyamoya Disease.

Modality *	Primary Role	Key Strengths	Main Limitations	Preferred Clinical Use
DSA	Structural reference	Highest spatial resolution, staging	Invasive, radiation	Diagnosis confirmation; staging; surgical planning
MRI/MRA	Structural and functional	Noninvasive, multi-parametric	Availability	Initial diagnosis; follow-up; parenchymal assessment
ASL MRI	Perfusion	No contrast or radiation	Lower spatial resolution	Hemodynamic assessment; follow-up
CTA	Vascular anatomy	High sensitivity for small vessels	Radiation	Diagnosis (when MRI unavailable); acute setting
CT Perfusion	Hemodynamics	Accessible, functional	Contrast use	Hemodynamic assessment; CVR estimation
SPECT	Perfusion and reserve	Functional reserve	Radiation	Hemodynamic assessment; ischemic risk stratification
TCD/TCCS	Hemodynamics	Bedside, low cost	Operator dependent	Screening; follow-up
P-VEP	Functional screening	Low cost	Limited specificity	Posterior circulation assessment

* ASL: Arterial Spin Labeling; CTA: Computed Tomography Angiography; CT: Computed Tomography; DSA: Digital Subtraction Angiography; MRI: Magnetic Resonance Imaging; MRA: Magnetic Resonance Angiography; P-VEP: Pattern-Reversal Visual Evoked Potentials; SPECT: Single-Photon Emission Computed Tomography; TCCS: Transcranial Color-Coded Sonography; TCD: Transcranial Doppler.

**Table 2:** Reported Diagnostic Accuracy by Modality (Selected Studies).

Modality *	Sensitivity (%)	Specificity (%)	Reference
CE-HR-MRI	85	86	[15]
7.0 T MRI/MRA	100	93	[16]
CTP-SIS	71	99	[31]
SPECT RCBF	100	97	[35]
CTA MDCT	80	90	[34]

* CE-HR-MRI: Contrast-Enhanced High-Resolution Magnetic Resonance Imaging; CTP-SIS: Computed Tomography Perfusion Source Images; MDCT: Multidetector Computed Tomography; RCBF: Relative Cerebral Blood Flow.

## Data Availability

No new data were created or analyzed in this study.

## Supplementary Materials

The following supporting information can be downloaded at: https://www.mdpi.com/article/10.3390/jcm15062410/s1, Table S1: PRISMA checklist.
